# The Importance of Mouse Models to Define Immunovirologic Determinants of Progressive Multifocal Leukoencephalopathy

**DOI:** 10.3389/fimmu.2014.00646

**Published:** 2015-01-05

**Authors:** Elizabeth L. Frost, Aron E. Lukacher

**Affiliations:** ^1^Immunology and Molecular Pathogenesis Graduate Program, Emory University, Atlanta, GA, USA; ^2^Department of Microbiology and Immunology, The Pennsylvania State University College of Medicine, Hershey, PA, USA

**Keywords:** progressive multifocal leukoencephalopathy, JC virus, natalizumab, mouse model, polyomavirus, tissue-resident memory T cells

## Abstract

Progressive multifocal leukoencephalopathy (PML) is a severely debilitating and often fatal demyelinating disease of the central nervous system (CNS) in immunosuppressed individuals caused by JC polyomavirus (JCV), a ubiquitous human pathogen. Demyelination results from lytically infected oligodendrocytes, whose clearance is impaired in the setting of depressed JCV-specific T cell-mediated CNS surveillance. Although mutations in the viral capsid and genomic rearrangements in the viral non-coding region appear to set the stage for PML in the immunosuppressed population, mechanisms of demyelination and CNS antiviral immunity are poorly understood in large part due to absence of a tractable animal model that mimics PML neuropathology in humans. Early studies using mouse polyomavirus (MPyV) in T cell-deficient mice demonstrated productive viral replication in the CNS and demyelination; however, these findings were confounded by spinal cord compression by virus-induced vertebral bone tumors. Here, we review current literature regarding animal models of PML, focusing on current trends in antiviral T cell immunity in non-lymphoid organs, including the CNS. Advances in our understanding of polyomavirus lifecycles, viral and host determinants of persistent infection, and T cell-mediated immunity to viral infections in the CNS warrant revisiting polyomavirus CNS infection in the mouse as a bona fide animal model for JCV-PML.

## Introduction

The human JC polyomavirus (JCV) persists silently in >50% of the healthy adult population, with recent evidence suggesting an even higher prevalence (1, 2). Seroepidemiological studies indicate that individuals are first exposed to JCV in late adolescence (3). Based on detection of JCV in tonsils and sewage, the virus is likely acquired via respiratory and/or fecal-oral transmission routes (4–6). JCV was discovered in 1971 as the etiologic agent of progressive multifocal leukoencephalopathy (PML) (7), a life-threatening demyelinating disease of the central nervous system (CNS) resulting from lytic infection of oligodendrocytes (8, 9). PML was first described in 1958 in patients with chronic lymphocytic leukemia and Hodgkin’s lymphoma (10), and has since been diagnosed in individuals immunosuppressed by a variety of hematological malignancies. Before the advent of highly active antiretroviral therapy (HAART), approximately 5% of individuals afflicted with HIV/AIDS developed PML, such that PML became regarded as an AIDS-associated disease (11). Profound immunosuppression, however, is not an essential prelude to PML. PML is seen in HIV-negative individuals with occult or minimal immunosuppression caused by old age, chronic liver or kidney disease, untreated dermatomyositis, and idiopathic CD4^+^ or CD8^+^ lymphopenia (12). No effective anti-JCV agents are currently available, and the prognosis for PML is poor (13). Recently, PML has emerged in patients receiving humoral immunomodulatory agents for autoimmune diseases and inflammatory disorders.

In 2005, a trilogy of articles in the *New England Journal of Medicine* described PML in patients with relapsing-remitting multiple sclerosis (MS) and Crohn’s disease given the monoclonal antibody natalizumab (Tysabri^®^) (14–16). Recent studies report that the risk of PML increases with duration of natalizumab therapy and is as high as 11.1 cases per 1000 patients in MS patients seropositive for JCV, >24 months of the monthly infusion therapy, and a history of immunosuppression (17). Most MS treatment regimens were designed to reduce autoreactive immune responses. Natalizumab is a humanized antibody against α_4_ integrin (CD49d), which complexes with the integrins β_1_ (to form very late antigen-4, VLA-4) or β_7_ on the surface of activated T cells (18). VLA-4 and α_4_β_7_ enable T cells to traffic to sites of infection/inflammation or to mucosal tissues, respectively. VLA-4 and α_4_β_7_ are required for T cell extravasation by mediating leukocyte arrest at activated vascular endothelium expressing the VLA-4 ligand vascular cell adhesion molecule 1 (VCAM-1) or the α_4_β_7_ ligand mucosal addressin cell adhesion molecule (MAdCAM-1). Supporting the hypothesis that natalizumab-mediated VLA-4 blockade impairs CNS immune surveillance is an early study showing that a cohort of 23 MS patients receiving natalizumab had decreased counts of CD4^+^ and CD8^+^ T cells, CD19^+^ B cells, and CD138^+^ plasma cells in the CSF compared to 35 untreated MS patients and 16 patients with other neurological diseases. Of 14 patients available for six-month followup after cessation of natalizumab therapy, all but one had decreased counts in each of these lymphocyte populations; the one exception being a patient having a modest elevation in CD4^+^ and CD8^+^ T cell counts coincident with a clinical MS replase (19). Other monoclonal antibody-based immunomodulatory therapies, including efalizumab (anti-LFA-1, for severe plaque psoriasis) and rituximab (anti-CD20, for B cell lymphoma), have been shown to put patients at risk for PML; because of this risk, efalizumab was taken off the market despite its efficacy in reducing rejection in kidney transplant recipients (20). With development of more intense steroid-avoidance immunosuppressive agents in transplantation medicine, there is concern that the incidence of PML may also rise in this patient population, as has happened for BK virus (BKV)-associated nephropathy. Recent evidence suggests that inadequate T cell-mediated surveillance for JCV-infected CNS glial cells is the common mechanism among immunosuppressive regimens conferring susceptibility for PML. As discussed above, natalizumab interferes with trafficking of circulating effector T cells into the CNS. Rheumatoid arthritis patients receiving rituximab show marked T cell depletion, particularly in the CD4^+^ T cell compartment, which is associated with clinical response (21). B cell-depletion therapies, such as rituximab, may affect T cell responses by depleting B cell-derived cytokines/chemokines, by eliminating B cells in their capacity as professional antigen-presenting cells to activate CD4^+^ T cells, or, possibly, by limiting availability of immune complexes to cross-present antigens to CD8^+^ T cells by FcγR^+^ dendritic cells (22). Immune reconstitution by HAART for AIDS or plasma exchange for monoclonal antibody therapy is the recommended treatment option for PML (23). Such regimens predispose patients to a rapid, robust, and often fatal influx of circulating leukocytes into the CNS termed immune reconstitution inflammatory syndrome (IRIS); paradoxically, these treatments can accentuate PML lesions, cause relapse of autoimmune disease or, in the case of organ transplant recipients, lead to graft rejection (24).

In addition to underlying depressed or altered immune function, viral determinants may also increase PML risk and/or disease severity. Mutations resulting in single amino acid substitutions in the host cell receptor binding domain of the viral capsid protein VP1 and rearrangements/deletions in the non-coding control region (NCCR) were found in most JCV sequences from cerebrospinal fluid (CSF) of PML patients (25–28). These mutations may constitute necessary viral determinants for PML and underlie the sharp discrepancy between the high prevalence of JCV infection and the low incidence of PML. Recent work demonstrated that rearranged NCCRs conferred increased early viral gene expression and DNA replication capability in glial cells (29). An important role for the JCV capsid protein in CNS tropism is supported by evidence that a hybrid virus containing the early genes of the monkey polyomavirus, SV40, and the late genes of the PML-JCV (Mad-1 strain) acquired the more restricted host range of JCV; i.e., the ability to infect human fetal glial cells but not monkey cells, and to hemagglutinate human type O red blood cells (30). Use of JCV VP1 virus-like particles (VLPs) further suggested that the PML-associated JCV capsid mutations alter viral tropism, retaining virion binding specificity for CNS glial cells but not to other non-CNS cell types, and differing from wild type VLPs in glycan specificity (25). However, pseudoviruses with these VP1 mutations failed to transduce glial cells, raising the possibility that these CSF VP1 variants are non-infectious (31). Whether these VP1 variants confer neurovirulence to JCV, and whether JCV acquires these VP1 mutations in the periphery or after entry into the brain, remain to be determined.

Several lines of evidence suggest that humoral immunity selects VP1 mutant polyomaviruses. Exposure of a library of VP1-mutagenized SV40 variants to a neutralizing monoclonal antibody selected viruses with mutations in solvent-exposed loops of VP1 that were resistant to neutralization by this antibody (32). BKV serotypes have been shown to vary in their level of cross-recognition by neutralizing antibodies generated by VLP immunization (33). Interestingly, BKV isolates from kidney transplant patients with nephropathy and viremia also had a high frequency of VP1 substitutions (34). These findings raise the possibility that VP1-specific neutralizing antibody responses select variant polyomaviruses with mutations in VP1 that enable escape from antiviral humoral immunity. Whether T cell immunosuppression/-modulation favors neutralizing antibody-driven selection of such polyomavirus escape variants is unknown.

Progress in understanding pathogenesis of JCV-induced PML and developing effective therapeutic approaches is handicapped by the low number of PML cases, inadequate understanding of risk factors (with only three broad risk factors described to date for natalizumab-treated MS individuals – JCV seropositivity, prior immunosuppression, and >2-years of therapy), and heterogeneity among PML patients (e.g., differences in immunosuppression regimens, HLA type, age, and gender). Additional obstacles include consistent patient compliance in clinical studies, under-reporting and under-recognition of the disease, and the rapidity of disease progression following diagnosis (35). Because JCV replicates only in humans, we have limited understanding of the pathogenesis of PML and the immune mechanisms needed to keep persistent polyomavirus infection in check. Studying the evolution of PML pathology rather than the endpoint of disease when PML is diagnosed is essential for identifying factors that predispose only a small fraction of immunosuppressed individuals and those receiving immunomodulatory therapy to PML.

An animal model of polyomavirus-induced CNS disease that mimics pathologic hallmarks of PML would circumvent these obstacles and enable us to address important unanswered questions, including:

1. Which facets of innate and adaptive immunity control JCV infection in the brain and how does immunosuppression/immunomodulation interfere with this control?
2. Are there circumstances in which antiviral immune surveillance in the CNS may prove pathological rather than protective?
3. Does the pathogenesis of PML vary with different immunomodulatory regimens?
4. How/when does JCV traffic to the CNS?
5. When are PML-associated viral mutations acquired and do they confer neurotropism/neurovirulence to JCV? Are these mutations the result of immune selection/evasion?
6. Why are individuals treated with humoral immune modulatory agents susceptible to JCV encephalitis but not to encephalitides caused by other persistent microbial pathogens (e.g., toxoplasmosis, HSV-1)?

A tractable small animal model of CNS infection by a natural host polyomavirus will provide insight into PML risk factors, mechanisms of disease, and provide a preclinical model to evaluate candidate antiviral agents. Here, we review current literature on T cell-mediated control of viral infections in non-lymphoid organs, including the CNS, describe potential mechanisms to dampen T cell function in the setting of persistent CNS infection, and advocate application of the mouse polyomavirus (MPyV) model to understand immune control of polyomavirus infection in the CNS.

## Evidence That JCV-Specific T Cells Confer Protection from PML

Effective immunity to viruses typically depends on CD8^+^ T cells and their ability to directly target and kill virally infected cells. Accordingly, presence of detectable JCV-specific CD8^+^ T cells in peripheral blood correlates with improved prognosis and survival in PML patients (36–38), whereas anti-JCV humoral responses do not (39, 40). In HIV^+^ patients, a detectable level of JCV-specific CD8^+^ T cells was coincident with a higher number of CD4^+^ T cells (41), the presence of which in peripheral blood has been positively correlated with PML survival (42). The dominant HLA-A2-restricted CD8^+^ T cell epitopes found in JCV-seropositive individuals are directed to determinants corresponding to VP1 residues 100–108 (p100) and 36–44 (p36), with the former being the dominant specificity (43). Staining with HLA-A*0201-VP1p36 and -VP1p100 tetramers showed that these JCV-specific CD8^+^ T cells have an effector-memory phenotype (CD62L^lo^CD45RA^−^CD49d^hi^) and can be found in the PBMCs of healthy individuals, perhaps contributing to the overall low incidence of PML (44, 45). Indeed, when measured early after PML diagnosis, the presence of JCV-specific CD8^+^ PBMCs predicted control of PML, while the absence of these cells predicted active PML progression (41). In HIV^+^ PML patients, CD8^+^ T cells can be found infiltrating the brain and co-localizing with infected oligodendrocytes at the edges of PML lesions (46) where the T cell receptor ligands MHC I and II are upregulated (47). Taken together, these findings suggest that JCV infection is predominantly controlled by CD8^+^ T cells.

Studies of human JCV-specific T cell responses have been predominantly based on analysis of PBMCs. Because few JCV-specific cells can be isolated from healthy individuals and PML patients, JCV-specific T cells are generally subjected to extended expansion and selection in tissue culture, which may obscure conclusions regarding their *in vivo* phenotype and function, as highlighted by an early study showing long-term *in vitro* T cell proliferation can profoundly underestimate frequencies of antigen-specific T cells *in vivo* (48). Although the development of MHC I tetramers for detecting JCV-specific CD8^+^ T cells has improved quantification of these cells, the low incidence of PML coupled with few defined HLA class I-restricted JCV epitopes limits direct analyses of JCV-specific CD8^+^ T cells in PML patients. Additionally, analysis of CNS-infiltrating T cells is hampered because PML brain lesions show minimal inflammation, which may be due to the patient’s immunosuppressive state and to the late stage of disease at time of diagnosis. Only one study has analyzed CNS-infiltrating JCV-specific T cells directly *ex vivo* by flow cytometry using a fresh brain biopsy of a natalizumab-treated MS patient with a pronounced T cell infiltrate secondary to IRIS (49). Examination of immune surveillance prior to diagnosis of PML is limited to CSF samples, which does not necessarily reflect immune infiltrates in the brain parenchyma (19). Given the limited data available regarding the type, function, and location of cellular infiltrates in the brain parenchyma, little is known about the status of immune surveillance in the CNS for JCV-infected cells prior to PML and during its progression.

Insights into the evolution and maintenance of JCV-specific T cell responses in the human CNS would greatly benefit from a mouse model of polyomavirus CNS infection. Use of this animal model would provide insight into the kinetics of anti-polyomavirus immune surveillance in the CNS, how immune suppression alters this surveillance and the incidence of neuropathology. Inbred strains of mice simplify identification of viral peptide T cell epitopes, which is essential to monitor the magnitude, phenotype, and function of virus-specific T cells. Incisive identification of determinants of effective CNS immune responses can be achieved using transgenic mice, mice with targeted genetic deletions, and antibody-mediated blockade of key interactions or deletion of specific cell types. Mouse models of viral infections can be optimized for pathogen dose and time postinfection to yield higher numbers of immune cells to study directly *ex vivo*. Furthermore, immune cells can be isolated from mouse tissues, providing insight into potential differentiation/regulation of cells *in situ* in the CNS. Such studies using mouse models are underpinned by a newfound appreciation that circulating T cells are phenotypically and functionally distinct from those resident in non-lymphoid tissues.

## CNS-Resident CD8^+^ Memory T Cells

Memory CD8^+^ T cells are heterogeneous in phenotype, differentiation, and function; these parameters are linked to their migration patterns and anatomic location (50). Since the original description of non-lymphoid organ-homing “effector memory” versus lymphoid organ-homing “central memory” populations (51), evidence is quickly accumulating that memory T cell heterogeneity is integrated with tissue residence; i.e., depending on their tissue localization, memory T cells vary in expression of chemokine receptors, adhesion molecules, and effector capabilities (52). Effector-memory T cells are now thought to be comprised of circulating and non-circulating subsets. The latter “tissue resident” memory T cells or T_rm_ cells are distinguished from circulating effector-memory cells by upregulation of CD69 and granzyme B (canonically indicative of TCR activation and cytotoxic effector capability, respectively) and cell surface expression of the α_E_(CD103)β_7_ integrin. Because the α_E_β_7_ complex binds to E-cadherin, CD103 expression implicates a role for these integrins in T cell retention in epithelium. Recent work showed that CD103 is variably expressed by CD8^+^ T_rm_ cells in different tissues, suggesting that CD103 *per se* is not a signature T_rm_ cell marker (53). CD69 directly antagonizes expression of S1P_1_, a sphingosine-1-phosphate receptor expressed by T cells to enable their egress from peripheral lymphoid organs (54); S1P_1_ downregulation is essential for retention of T cells in tissues (53). TGF-β is a key mediator of CD103 expression by activated CD8^+^ T cells in the skin and intestinal mucosa, as well as the CNS, and TGF-β may also be involved in upregulating CD69 (55–57). CD8^+^ T_rm_ cells persist long-term in the skin and in mucosal sites such as the respiratory tract, female reproductive tract, and gut (58–61), and for the intestine at least, maintenance of CD8^+^ T_rm_ cells is antigen-independent (56). A rapidly expanding body of literature demonstrates that T_rm_ cells contribute to host defense to bacterial and viral reencounter at mucosal and epidermal sites (62, 63).

Antiviral CD8^+^ T cells also establish residence in the CNS but appear to differ in their requirements for function and survival compared to those in extra-CNS non-lymphoid tissues. Intracranial (i.c.) inoculation of mice with vesicular stomatitis virus (VSV), an acutely infecting pathogen, resulted in the progressive accumulation in the brain of CD103^+^ CD69^hi^ granzyme B^hi^ virus-specific CD8^+^ T_rm_ cells, whose maintenance was antigen-independent and required CD103 (64). Differences in virus interactions with their hosts (e.g., levels of antigen persistence, cytopathic/non-cytopathic cell fate and host cell tropism) will undoubtedly influence the establishment, maintenance, and function of CNS-resident memory T cells and their immunosurveillance efficacy for infected cells.

The presence of CNS-infiltrating CD8^+^ T cells circumscribing demyelinated lesions was associated with improved clinical outcome in HIV/AIDS-related PML patients (46). This association has been extended to explain the development of PML in MS patients receiving natalizumab. MS progression is characterized by two distinct phases: a primary relapsing-remitting stage in which repetitive and acute infiltration of the brain by myelin-reactive T cells occurs and inflammation resolves; and a secondary progressive phase in which little inflammation is observed but lesions of demyelination and functional disability worsen (65). Because natalizumab acts to exclude circulating activated T cells from the CNS, it is possible that no JCV-specific T cells infiltrate the brains of natalizumab-treated MS patients with active JCV replication. MS is a difficult disease to diagnose, and patients likely experience acute autoreactive inflammation prior to initiation of natalizumab therapy. Myelin-reactive inflammation may render the blood–brain barrier “permeable” to JCV (as free virions or via infected cells, as discussed below) as well as JCV-reactive T cells. In this connection, it merits noting that natalizumab is typically not the first-line treatment option for MS. If JCV infection in the brain and a subsequent T cell infiltration occurred prior to the administration of natalizumab therapy, CNS-infiltrating JCV-specific cells would not be affected and could potentially establish a resident memory population. Based on data from various animal models of tissue resident memory T cells, in the context of persistent infection in the CNS functional JCV-specific T_rm_ cells might survive long-term or be driven to dysfunction and deletion. In the former case, JCV-specific T_rm_ cells would be protective, whereas in the latter situation, exhausted T_rm_ cells may predominate and fail to limit the number of infected oligodendrocytes. Natalizumab, then, would prevent replenishment of CNS-infiltrating JCV-specific T cells from circulating blood and result in deficient T cell-mediated immune surveillance for infected glial cells. These alternate scenarios may help explain the rarity of PML even in immunocompromised individuals. Knowing the longevity of functional T_rm_ cells in the CNS, which would be most readily studied using a mouse model of polyomavirus CNS infection, may help explain the increased risk of PML with long-term natalizumab therapy.

## The Inhibitory PD-1 Receptor Balances Immunity and Immunopathology in the CNS

The brain is widely considered to be an “immune-privileged” organ. Until recently, immune privilege was thought to result from a complete exclusion of the immune effector cells; however, healthy immune-privileged sites are actually subject to active immune surveillance, requiring the functionality of immune effector cells be tightly managed to protect vital, non-renewable tissues (66). Regulating cells of the immune response typically depends on the balance of activating and inhibitory signals. PD-1 (CD279), a CD28-family molecule, is expressed by activated T cells and counters the activation signaling cascade initiated by TCR ligation. PD-1 expression is significantly elevated on JCV-specific CD4^+^ and CD8^+^ T cells from peripheral blood of PML patients (67). PD-1 is induced by TCR signaling and its expression is maintained in settings of persistent antigenic stimulation such as chronic viral infection, cancer, and autoimmunity. PD-1 binds to two ligands, PD-L1 (also called B7-H1; CD274) and PD-L2 (CD273) that differ in expression patterns: PD-L1 is broadly expressed by hematopoietic and parenchymal cells; and PD-L2 is inducibly expressed on DCs and macrophages (68). Recent work indicates that variable levels of PD-1 signaling translates to differentially dampening T cell functions, with low PD-1 levels inhibiting TNF-α, IL-2 production, and cell proliferation and higher levels inhibiting cytotoxicity and IFN-γ production (69). Sustained high PD-1 expression in the setting of chronic viremic infection mediates exhaustion of virus-specific CD8^+^ T cells (70).

Although PD-1 regulation of T cells has been intensively investigated for systemic persistent viral infections, surprisingly little is known about its role in viral encephalitis. In mouse cytomegalovirus encephalitis, brain-localized CD8^+^ T cells express PD-1, activated microglia and astrocytes express its ligand (PD-L1), and PD-1 blockade in microglia/astrocytes and CD8^+^ T cell co-cultures increases IFN-γ production (71). CD8^+^ T cells infiltrating the CNS of mice persistently infected by the gliatropic mouse JHM coronavirus are also PD-1^hi^. IFN-γ receptor signaling by oligodendrocytes induces their expression of PD-L1 (72), which, in turn, limits effector activity of PD-1^hi^ JHM-specific CD8^+^ T cells. Engagement of these PD-1^hi^ cells with PD-L1^+^ oligodendrocytes prevents T cell-mediated axonal bystander damage, but does so at the cost of negating viral clearance (73, 74). Interestingly, CNS infection by VSV, a neurotropic and non-demyelinating pathogen, is associated with robust virus-specific CD8^+^ T cells brain infiltrates, but these T cells lack PD-1 expression (64). PD-1 is elevated on CD8^+^ T cells from PML patients (67). These studies raise the possibility that PD-1 upregulation is a property of T cells responding to gliatropic viral infections. These data are in line with the concept that PD-1 inhibits T cell-mediated immunopathologic demyelination, a concept supported by studies documenting a protective role for PD-1 in the experimental autoimmune encephalomyelitis (EAE) mouse model of MS (75–78).

Because persistent polyomavirus infection establishes a low-level antigen setpoint, PD-1 upregulation by polyomavirus-specific T cells is unexpected, and suggests that sustained strong TCR signaling may not be essential for PD-1 expression in the brain. In HIV-positive individuals, PD-1 expression is higher on T cells in the CSF than those in blood, despite viral RNA levels being lower in the CSF (79). Certain common γ-chain cytokines (IL-2, IL-7, IL-15, and IL-21) and type I IFNs have been shown to induce and maintain PD-1 expression on TCR-activated cells (80–82); and type I and type II IFNs, IL-6, IL-2, IL-7, and IL-15 upregulate PD-L1 (83). With regard to MPyV, IFN-β transcripts have been shown to be elevated in brains of mice given MPyV i.c. (84). This raises the possibility that the polyomavirus-induced proinflammatory environment in the CNS could complement low/intermittent TCR engagement to upregulate PD-1 on antiviral T_rm_ cells in the brain. A polyomavirus CNS infection animal model would enable investigation of the mechanisms of PD-1 upregulation by brain-resident, virus-specific T cells and the functional role(s) of PD-1 expression by T cells in the CNS.

## Using JCV to Model PML in Animals

A tractable animal model of PML requires sufficient similarities between hallmarks of disease in humans, including cell targets for viral replication, mechanisms of immune control, associated risk factors, and neuropathology. Until recently, however, attempts to model PML in mice utilizing the etiologic agent of PML, JCV, have been handicapped by the tight species specificity of *Polyomaviridae*. Lacking a viral DNA polymerase, polyomaviruses rely on the host cell DNA polymerase apparatus to replicate their genomes, and thus have devised strategies to override cell cycle checkpoints (85). The species restriction for polyomaviruses is controlled at the level of binding by the host cell DNA polymerase α-primase complex to the viral origin of replication (86, 87), a molecular interaction reflective of the co-evolution of each *Polyomaviridae* family member with a particular vertebrate species. Non-productive infection by polyomaviruses can result in the integration of the viral genome into the host chromosomal DNA resulting in tumor formation (88–90). Accordingly, experiments involving i.c. inoculations of non-human primates and mice with JCV resulted in non-productive infection and development of astrocytomas or glioblastomas but not PML-like disease (91–96). Transgenic mice containing the early region of JCV in all cells predominantly expressed T antigens in oligodendrocytes and exhibited a dysmyelination phenotype, but did not recapitulate the demyelination associated with JCV encephalitis in human (97). These findings have largely obviated the use of JCV infection in unmanipulated mice to model PML.

To partially preserve the species specificity of JCV infections in animal hosts, attempts have been made to use JCV in humanized mouse models. Low levels of JCV DNA were detected in the urine and blood of JCV-infected NOD/SCID/IL-2Rα^−/−^ mice reconstituted with human fetal bone marrow, thymus, and liver, as were low numbers of IFN-γ-producing T cells (98). In the absence of human CNS tissue to provide cellular targets for JCV replication, this system cannot recapitulate PML. This obstacle has recently been overcome. Goldman and colleagues created human glial chimeric mice by engrafting human bipotential oligodendrocyte-astrocyte progenitor cells into congenitally hypomyelinated *Rag2^−/−^ Mbp^shi/shi^* neonatal mice, resulting in efficient colonization of the mouse brain with human astrocytes and oligodendrocytes and myelination (99, 100). Using these human glial chimeric mice, Kondo et al. recently reported that intracerebral delivery of Mad-1 JCV resulted in early widespread productive infection of the engrafted human astrocytes, focal demyelination, and gliosis (101). Only rare oligodendrocytes were infected early postinfection, but at late timepoints large numbers of T antigen^+^ VP1^−^ oligodendrocytes were detected. These findings raise the provocative possibility that demyelination may in large part be accounted for by deficient trophic support for oligodendrocytes resulting from death of productively infected astrocytes rather than by elimination of oligodendrocytes by lytic JCV infection. Another notable finding in this study was the rapid emergence of a sizeable number of VP1 genomic mutations, with at least two previously seen in JCV isolates from PML patients. Thus, astrocytes may be a major site for emergence of neurovirulent VP1 variant viruses. This chimeric mouse model represents an important advance toward understanding mechanisms of pathogenesis of demyelination; however, infection of these human glia-engrafted mice cannot provide insight into the role of JCV-specific immune responses in the CNS.

## JCV Entry to the CNS

A major unresolved question is the mode of transit of JCV to the CNS; i.e., as free virus and/or via infected “Trojan Horse” cells. Deep sequencing of JCV NCCR in matched urine, plasma, and CSF samples from a PML patient provides strong support for a hematogenous route for viral dissemination (102), a conclusion in line with earlier evidence that VP1 mutations are detected in blood and CSF, but not in urine, of PML patients (27). Human brain microvascular endothelial cells have been demonstrated to be permissive for JCV infection, a finding supporting the possibility that JCV may cross the blood–brain barrier by infecting endothelial cells (103). In favor of cell-based blood-to-brain carriage is an early study reporting detection of JCV and BKV DNA in peripheral blood leukocytes from healthy adults (104). Subsequent work has focused attention on B cells and/or CD34^+^ hematopoietic progenitor cells (HPCs) as candidate vehicles for conveying JCV to the CNS. JCV DNA, as well as expression of T antigen and VP1, has been observed in HPC cell lines, B cell lines and primary B cells infected *in vitro* by high-dose virus inocula, and rare JCV DNA^+^ B cells have been detected in PBMCs from a PML patient (4, 105). In addition, the rearranged NCCR contains multiple binding sites for the Spi-B and NF1-X, transcription factors that enhance JCV replication and are expressed by B cells, HPCs, and glial cells [reviewed in Ref. (13)]. Notably, gene expression of unfractionated blood, and sorted CD19^+^ B cells, and CD34^+^ HPCs from MS patients receiving natalizumab revealed upregulation of genes involved in B cell activation and differentiation, including Spi-B (106, 107). These findings are in line with a proposal that upregulation of specific transcription factors that bind JCV NCCR underlie a resurgence of JCV replication in natalizumab-treated individuals (108, 109). Because B cells are endowed with the recombination apparatus enabling V(D)J recombination of immunoglobulin gene segments, B cells have also been proposed to provide an environment conducive for JCV genome recombination and/or rearrangement (13), despite the absence of RAG-dependent recombination signal sequence motifs in the JCV genome. Whether B cells are truly capable of supporting JCV replication remains to be demonstrated, particularly in light of recent data suggesting that B cells may carry intact input JCV virions and transfer them to susceptible glial cells (110). CD49d antibody-mediated blockade in mice and non-human primates is associated with elevated circulating CD34^+^ cells, an observation recapitulated in natalizumab-treated MS patients (111–113). In humans, natalizumab has also been reported to mobilize CD34^+^ cells, pre-B cells, and B cells into the circulation (114, 115). These observations give additional impetus to the value of an immunocompetent mouse model of PML to define the cellular vehicle by which JCV is transported to the brain parenchyma, the status of JCV replication in these cells, and investigating the possible role of VLA-4 blockade in promoting JCV spread to the CNS.

## MPyV as a Model to Understand Human Polyomavirus Pathogenesis

Mouse polyomavirus, the founding member of the *Polyomaviridae* family, is structurally and genomically similar to JCV, BKV, and SV40 polyomaviruses. All polyomaviruses consist of a double-stranded, covalently closed circular ~5-kb DNA genome encapsidated by a non-enveloped icosahedral shell composed of 72 pentameric VP1 capsomers. The genomes of all polyomaviruses have a ~500-bp NCCR containing the origin of replication bidirectional promoters separating the genome into early and late genes, with respect to the onset of viral DNA synthesis: an early region encoding the non-structural small T and large T antigens; and a late region encoding the viral capsid proteins VP1, VP2, and VP3. Unlike JCV, the MPyV genome does not encode an agnoprotein in its late region and contains an additional early region sequence encoding the non-structural middle T antigen, which mediates cellular transformation and tumor induction (116).

Mouse polyomavirus also resembles BKV and JCV with regard to infectivity and interaction with the immune system. Epidemiologic surveys of wild mice showed that MPyV, like its human counterparts, is a widely prevalent, harmless pathogen in its natural host reservoir (117, 118). MPyV infects a variety of epithelial and mesenchymal cells (119), macrophages, and DCs, but not lymphocytes (120). Neuroectodermal lineage cells were stated to be non-permissive for MPyV replication (119), but evidence for this host cell range restriction is lacking. As described above for PML-JCV variants, strains of MPyV carrying single amino acid differences in VP1 differed in glycan specificity, which in turn altered tissue tropism and pathogenesis (121, 122). Similar to reports of long-term persistence of JCV and BKV DNA in a variety of human tissues (6, 109, 123), MPyV DNA has been detected in multiple organs, including those of the CNS, kidney, and bone marrow, for months after acute infection in both immunodeficient and immunocompetent mice (124–126), with decline in immunologic status setting the stage for viral reactivation. Both human and mouse polyomavirus infections elicit potent neutralizing antibody responses directed toward VP1 that inhibit capsid binding to sialyated glycolipid and glycoprotein receptors on host cells. MPyV persistently infects mice in the presence of virus-neutralizing VP1 Abs (127). Similarly, neutralizing Abs against JCV, typically present in most individuals, confer no protection against PML (128). While neither human nor mouse polyomaviruses cause overt disease in immunocompetent adult hosts, immunosuppression provides an opportunity for both human and mouse polyomaviruses to induce a variety of disease processes (129). MPyV-induced rejection of mouse renal allografts has been used to understand how immunosuppression alters the evolution of polyomavirus-associated nephropathy and how the immune response to polyomavirus infection contributes to allograft injury (130, 131).

Mouse polyomavirus replication causes disease in the CNS. Primary glial cells derived from mouse corpus callosum showed that type 1 astrocytes, but neither type 2 astrocytes nor oligodendrocytes, were infected by MPyV (132). This was not the case *in vivo* as infection of congenitally athymic mice with MPyV resulted in wasting disease and spinal cord demyelination consequent to infection of oligodendrocytes (133). Demyelinated lesions were not observed in euthymic mice, emphasizing the role of immune suppression in disease progression. Infected nude mice eventually developed hind limb paralysis, which was attributed to vertebral bone tumors rather than PML-like disease (134, 135). In each of these early studies, mice were infected by a natural route of transmission via contamination from a neighboring mouse room. In a study involving i.c. inoculation of adult nude mice with the LID strain of MPyV, which caused fatal kidney and brain hemorrhages in newborn C3H mice (122), paralysis and vertebral tumors developed in the absence of demyelination (135). This publication was largely responsible for the *de facto* moratorium on use of MPyV CNS infection as a model for PML. Interestingly, molecular modeling showed that the valine-to-alanine substitution at VP1 amino acid 296 in LID was orthologous to ^269^VP1 of JCV, where a serine to phenylalanine/tyrosine mutation was among the frequent mutations detected in VP1 genes of JCV isolates from PML patients (26). Although an MPyV-mouse model of CNS disease cannot reproduce all aspects of JCV-PML pathology in humans, just as significant aspects of other mouse models do not fully recapitulate human disease, new evidence for the existence of tissue-specific protective antiviral T cells and recent work from our laboratory using i.c. inoculation of MPyV in tumor-resistant mice, suggest that MPyV may prove to be an important animal model of polyomavirus-induced CNS demyelination.

## Conclusion

Progressive multifocal leukoencephalopathy, a rare complication of immunosuppression, is caused by infection of the CNS by JC virus, a highly ubiquitous and silent human pathogen in healthy individuals. This wide discordance between virus prevalence and disease incidence appears to stem from the coalescence of multiple predisposing factors including viral determinants that alter host cell tropism, host immune determinants that affect CNS surveillance for infected glial cells, and variability in the underlying immunosuppressive disease/treatment regimen. Lack of a tractable animal model due to the tight species specificity of *Polyomaviridae* has stymied efforts to determine the contributions of each factor to PML pathogenesis. Here, we have reviewed the literature describing previous attempts to develop animal models for PML and propose use of mouse polyomavirus to study the interplay between the host immune response and infection in the brain. Significant research using peripheral blood mononuclear cells and autopsy/biopsy tissue from PML patients implicates a role for JCV-specific T cell responses in disease outcome. The MPyV encephalitis model should provide insight into mechanisms of JCV-induced demyelination and evolution of protective/pathological immune responses to JCV CNS infection *in situ*, as well as provide a preclinical platform to evaluate strategies to prevent and control PML.

## Conflict of Interest Statement

The authors declare that the research was conducted in the absence of any commercial or financial relationships that could be construed as a potential conflict of interest.
